# Improving Compliance with Screening of Diabetic Patients for Microalbuminuria in Primary Care Practice

**DOI:** 10.1155/2013/893913

**Published:** 2013-10-09

**Authors:** Abeer Anabtawi, L. Mary Mathew

**Affiliations:** Department of Medicine, Unity Health System, 1555 Long Pond Road, Rochester, NY 14626, USA

## Abstract

Studies showed suboptimal compliance rate of primary care physicians with microalbuminuria screening. This study evaluated impact of electronic medical records (EMR) and computerized physicians reminders on compliance rate and showed small to modest improvement. Combining EMR with quality control monitoring has significantly improved compliance [OR 1.556, 95% CI 1.251–1.935, *P* = 0.006].

## 1. Introduction

Diabetic nephropathy develops in 20–40% of diabetic patients and is the leading cause of end-stage renal disease (ESRD) in the western world. The cost for treating diabetic ESRD exceeded $23 billion per year in USA [1, 2]. One of the earliest clinical markers is the appearance of low but abnormal levels (≥30 mg/day) of albumin in the urine. Persistent albuminuria in the range of 30–299 mg/24 h (microalbuminuria) if untreated can progress to macroalbuminuria (≥300 mg/24 h) with gradual decline in glomerular filtration rate and the development of chronic kidney disease (CKD) [3, 4]. Microalbuminuria is also a marker for increased cardiovascular risk in such patients [5]. 

Numerous guidelines for diabetic care recommend an annual urine screening test to assess albumin excretion. The American Diabetic Association recommends performing microalbuminuria screening in patients who have had type I diabetes for at least 5 years or in patients with type II diabetes at time of diagnosis [6]. However, studies demonstrated suboptimal compliance of primary care physicians (PCP) with these recommendations with variable compliance rate of 14–49% [7–9]. Several methods were suggested to improve PCP compliance at multiple levels including patient education and reminders, physician education, and continuous charts reviews for quality improvement and feedback [10]. Electronic medical records (EMR) enabled with physician reminder system have gained significant interest in recent years as a tool that is shown to improve compliance [10, 11]. However, the magnitude of this improvement has been variable, and its cost effectiveness remains controversial [12, 13]. This study evaluated the compliance rate of microalbuminuria screening after two years of introducing an EMR enabled with computer-generated reminder system for diabetes care guidelines. It also evaluated the impact of combining EMR with quality control monitoring in enhancing compliance.

## 2. Methods

### 2.1. Retrospective Analysis

#### 2.1.1. Patients

All patients with type II diabetes, who were registered at Unity Faculty Partners (UFP) primary care facility between January 2008, and December 2009, were included. Patients who were diagnosed with an established CKD stage IV or higher and have been followed by nephrologists were excluded. Patients who were known and/or treated for microalbuminuria were still included as per guidelines [6]. 

UFP clinics introduced EMR in October 2006 (NextGen EMR, Horsham, PA). Records are enabled with a computer-generated reminder that highlights the recommended screening tests and their due dates. These reminders are generated every time the patient electronic records are opened by a physician. The system, however, does not ask for response for these reminders before proceeding. The care of each patient at UFP is shared between an internal medicine resident and one of the six faculty member PCPs.

#### 2.1.2. End Points

**Step 1**

Two years after introducing EMR, a quality improvement (QI) project to evaluate microalbuminurea screening compliance rate was conducted. The results were disseminated to the PCPs and their residents.

**Step 2**

One year after Step 1, QI project was repeated to assess the combined effect of EMR and Step 1 intervention on the compliance rate of microalbuminurea screening.

#### 2.1.3. Statistics

SPSS (V15, Chicago, IL) was used for statistical analysis. Mantel-Hanzel test was used to calculate the odds ratio and 95% CI. *P* value of <0.05 is considered statistically significant.

#### 2.1.4. Results

A total of 259 diabetic patients were registered at UFP during the study period. Twenty-seven patients (10.4%) were excluded due to CKD ≥ stage IV. The remaining 232 patients (140 males, 92 females) had a median (interquartile) age of 61 years (52–72). Five of these patients were included only during Step 1, while 19 patients were included only during Step 2 due the date of leaving or joining UFP clinics.

In Step 1 [*n* = 213], microalbuminuria screening was ordered in 120 patients (56.3%). The test was completed in 101 of these patients (84.2%). In Step 2 [*n* = 227], the test was ordered in 158 patients (69.6%), and 134 of these patients completed the test (84.8%). Compliance with microalbumin screening significantly improved during Step 2 compared to Step 1 [OR 1.556, 95% CI 1.251–1.935, *P* = 0.006].

## 3. Discussion

Our compliance rate for microalbuminurea screening in Step 1 was 56.3%, higher than the quoted national average of 14–49%. Multiple factors may have contributed to this higher rate: EMR, computerized physicians reminders, and the setting of a teaching practice of a residency program. However, the study shows that even with the use of EMR and physicians reminders the compliance rate is still suboptimal with nearly half of the patients being excluded from the benefits of early detection of diabetic nephropathy. Similar findings were seen in some previous studies, and a recent Cochrane systematic review by Shojania et al. concluded that computerized point-of-care reminders have small to modest effect on the quality of health care [13, 14]. The review evaluated different features of computer reminders and found that the only feature that showed a trend toward larger improvement in compliance was the requirement for providers to enter a response to the reminder before being able to use EMR [13].

Several interventions have been evaluated to improve quality of diabetes care [10, 11, 14, 15]. These included professional interventions like physician's education, quality control through audits, EMR, and physicians' reminders, organizational interventions like regular follow-up schedule, patient appointment reminder system, and the availability of diabetes nurse practitioner, patient-oriented interventions like patient education and counseling. Previous studies evaluated different combinations of these interventions in comparison to the usual standards of care with no available studies directly comparing these interventions' efficacy to each other. A systemic review did not recognize one intervention to be superior to the others and concluded that multifaceted approach of different interventions is the effective way in improving quality of care [15]. The significant improvement in compliance rate in Step 2 of the current study emphasizes the importance of such multifaceted approach as EMR was combined with the QI process. EMR showed the advantage of easy data accessibility and monitoring. 

The patients' sample can be considered to be representative of the general population of diabetic patients; the types of interventions in this study including EMR, physicians' reminders, and combination with quality monitoring and audits are all applicable to the different settings of primary care practice. These factors may facilitate generalization of the study results. However, this study was conducted in a teaching primary care facility where internal medicine trainees are involved in patients care and in the audit process which should be considered when generalizing these results.
